# The effect of different borehole spacing and detonation time difference on crack propagation in double-hole shaped charge blasting

**DOI:** 10.1371/journal.pone.0341373

**Published:** 2026-01-20

**Authors:** Shixiang Xu, Bo Wu, Zhongsi Dou

**Affiliations:** 1 School of Civil and Architectural Engineering, East China University of Technology, Nanchang, China; 2 Engineering Research Center for Digital Risk Control of Underground Engineering of Jiangxi Province, East China University of Technology, Nanchang, China; Shenyang Jianzhu University, CHINA

## Abstract

Achieving precise through-crack between shaped charge blasting holes depends critically on the design parameters of the blasting operation. This study investigated the influence of borehole spacing and detonation time difference on crack propagation in shaped charge blasting using LS-DYNA numerical simulation. The reliability of the numerical model was verified through experiment test, and a double-hole shaped charge blasting calculation model was established. The dynamic evolution law of crack in double-hole shaped charge blasting under different borehole spacing and detonation time difference was analyzed. The results indicate that increasing borehose spacing leads to more severe stress wave attenuation, significantly reducing the peak effective stress at the center point between holes. Micro-delayed detonation regulates the stress field through timing control, thereby altering crack propagation behavior. As the delay time increases, crack originating from the first detonated hole account for a larger proportion of the propagation path, and the crack connection point shifts toward the later-detonated hole. Optimizing borehole spacing and micro-delay detonation can effectively promote inter-hole crack propagation. This study provides a theoretical basis for designing shaped charge blasting parameters in mining operations, tunnel excavation, and other engineering applications.

## 1. Introduction

The shaped charge blasting technology has gained widespread application in mining operations and tunnel excavation due to its precise directional energy control and superior rock fracturing effect. Through specialized charge structure design, this technology concentrates explosive energy release along predetermined direction. This approach not only significantly enhances energy utilization efficiency but also effectively minimizes disturbance and damage to surrounding rock, providing reliable technical support for precision blasting under complex geological conditions [1,2]. However, engineering practice shows that the formation of through-cracks between holes and the quality of fracture surfaces in shaped charge blasting depend on the synergistic optimization of key parameters such as borehole spacing and detonation time difference. Improper parameter selection can severely compromise blasting effectiveness [3–5]. Therefore, investigating the influence of borehole spacing and detonation time difference on the dynamic propagation behavior of crack is essential for optimizing shaped charge blasting design.

Borehole spacing is a critical blasting design parameter affecting stress wave superposition effect and crack propagation. Yang et al. [6,7] studied the propagation process of inter-hole crack generated by slotted blasting based on dynamic focal line experiment, analyzing the influence of parameters such as slot angle and depth on crack propagation to penetration. The detonation time difference affects the crack propagation by controlling the temporal distribution of the blasting stress field [8]. Tang et al. [9] conducted dual-hole bench blasting model test and found that increasing delay time shifts the strain concentration zone from horizontal to vertical, thereby altering the overall fragmentation pattern. Liu et al. [10] obtained through experiment and numerical simulation that under delayed initiation condition, the region of stress wave superposition shifts, promoting preferential crack propagation along the borehole connection line, with crack from the later-initiated hole extending further. Meng et al. [11] investigated the effect of delay time on rock mass damage under in-situ stress condition. They indicated that increasing inter-hole delay enhances vertical stress wave superposition, leading to increased secondary crack around later-detonated hole and a gradual shift of crack connection points toward these later hole. Qiu et al. [12] emphasized that a common blast pit can only form between holes when the initiation delay interval is shorter than the time required for new free surface formation. Hashemi and Katsabanis [13] used LS-DYNA numerical simulation to confirm the necessity of delayed initiation for crack development. However, it should be particularly noted that improper selection of initiation time difference can reduce crack penetration effectiveness [14–16].

Although research has made some progress in different borehole spacing and detonation time difference, studies on the dynamic evolution of inter-hole crack under these conditions in shaped charge blasting have yet to be conducted. This limitation hinders the widespread application of shaped charge blasting technology. To address the aforementioned research gaps, this study aims to different borehole spacing and detonation time difference on crack propagation between boreholes in shaped charge blasting. It seeks to reveal the underlying patterns of how different parameter combinations influence shaped charge blasting performance, thereby providing theoretical foundations and methodological support for optimizing shaped charge blasting design.

## 2. Mechanism of crack propagation between double-hole shaped charge blasting

**Fig 1:**
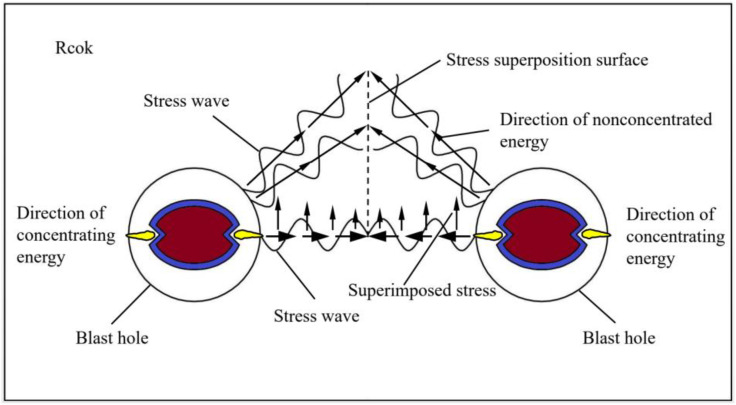
Mechanical model of shaped charge double-hole blasting.

The formation of through-crack between double-hole shaped charge blasting holes results from the combined effects of the shaped charge jet, explosion shock wave, stress wave, and explosive gas. Specifically, after the shaped charge detonates, a rapid chemical reaction occurs, the charge casing is subjected to intense compression and squeezing by the explosive gas, generating extremely high pressure in the shaped charge direction due to detonation, thereby forming a high-speed, high-density shaped charge jet. This jet impinges on the borehole wall, penetrating it to form an initial guiding crack. In the non-shaped charge direction, the presence of the shaped charge tube casing provides a certain degree of cushioning effect on the detonation product, reducing their direct impact on the borehole wall and inhibiting the propagation of crack in the non-shaped charge direction. The explosive stress wave generated by the shaped charge blasting and the quasi-static effects of the explosive gas promote further crack propagation. When the tensile stress at each point along the line connecting the two blast holes exceeds the tensile strength of the rock, the rock fails, forming a through crack along the line connecting the two blast holes. The mechanical analysis model of the double-hole shaped charge blasting is shown in Fig 1.

When two holes are detonated simultaneously, wave superposition generates twice the tensile stress at the superposition surface, intensifying tensile stress at the center of the two blast holes. Gas expands and compresses from both holes in the energy accumulation direction. According to wave interference principle, the mechanical calculation formula for the shaped charge direction is [17]:


$$\sigma_{t}=2\int_{0}^{\frac{L}{\text{2}}}x\int_{0}^{\frac{\pi }{\text{2}}}\sigma_{r}\sin\theta d\theta +\sigma_{qr}$$ (1)

In the formula: *r* is the distance from any point in the rock to the center of the blast hole; *L* is the spacing between blast holes; *σ*_*r*_ is the peak stress at any point in the rock; *σ*_*qr*_ is the pressure of the explosive gas.

When *R = 2 r*_*0*_ *+ L* is taken as the center distance between blast holes, *σ*_*r*_ is the peak stress at any point. The stress perpendicular to the radial direction between two blast holes decreases toward the center point of the line connecting the blast holes. At the center point, the stress can be superimposed to obtain *2σ*_*r*_*sin θ + σ*_*qr*_. As *L* increases, the propagation time of the explosive stress wave and explosive gas increases. The attenuation of the stress wave and explosive gas reduces the effectiveness of the superimposed stress field, thereby failing to effectively promote crack penetration.

## 3. Numerical solution reliability verification

### 3.1. Numerical model

A numerical analysis model was established in LS-DYNA software, using the MAT_HIGH_EXPLOSIVE_BURN model for the explosive material. The parameters of the explosive material are shown in Table 1.

**Table 1:** Explosive and parameters of equation of state.

*ρ* (g • cm^-3^)	*ν*_*D*_ (cm • μs^-1^)	*A* (GPa)	*B* (GPa)	*R*_1_	*R*_2_	*ω*	*E* (GPa)
1.3	0.4	214.4	0.182	4.2	0.9	0.15	4.192

The material properties of the energy gathering tube are characterized using the MAT_PLASTIC_KINEMATIC model to describe the mechanical changes. The mechanical parameters of the energy gathering tube are shown in Table 2.

**Table 2:** Mechanical parameters of PVC energy gathering tube.

Material	*ρ* (g • cm^-3^)	*G* (GPa)	*μ*	*σ* (GPa)
PVC	1.43	43	0.32	0.0617

The rock material was using the MAT_JOHNSON_HOLMQUIST_CNCRETE model, and the main parameters of the rock are shown in Table 3. The maximum principal stress failure of the rock is defined to analyze crack propagation.

**Table 3:** Main parameters of rock material.

Material	*ρ* (g•cm^-3^)	*μ*	*G* (GPa)	*f*_c_ (GPa)	*T* (GPa)	*A*	*B*	*C*	*N*	*D*_1_	*D*_2_
Rock	2.18	0.26	14.86	0.048	0.004	0.79	1.60	0.007	0.61	0.04	1

### 3.2. Explosion test of PMMA

PMMA is an ideal material for studying crack propagation in laboratory settings due to its transparency, which allows direct observation of crack morphology. Its fracture mechanics behavior is similar to that of brittle rock [18,19]. Xu et al. [20] conducted a shaped charge blasting test using PMMA. The energy gathering tube was made of rigid PVC plastic tubing, and the size of the PMMA specimen was 1200 mm × 600 mm × 12 mm. The blast hole was located at the center of the specimen with a diameter of 60 mm.

### 3.3. Comparison and analysis of result

**Fig 2:**
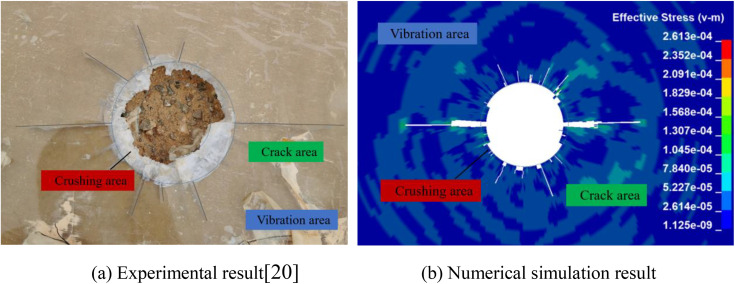
Comparison of experimental and numerical simulation result.

The dynamic propagation process and mechanical behavior of explosive crack were studied through the use of PMMA shaped charge blasting experiments. Given the complexity, difficulty in capturing and recording, and potential hazards of the blast process, this research employs numerical simulation methods to establish a numerical simulation model that aligns with the PMMA model experiment, in order to analyze the stress wave propagation process and crack dynamic evolution behavior of the shaped charge blasting. The comparison between the focused shaped charge blasting PMMA crack propagation experiment and the numerical simulation result is shown in Fig 2.

Under the action of the explosive shock wave, the dynamic compressive strength of PMMA is significantly lower than that of the shock wave, resulting in the formation of a crushing area around the blast hole. As the shock wave propagates outward, it gradually attenuates into a stress wave. At this stage, the PMMA develops circumferential tensile stress under the stress wave’s influence. When this stress reaches the tensile strength limit of the PMMA, radial tensile crack appear. Due to the formation of a focused jet along the shaped charge direction in blasting, this jet penetrates the borehole wall to create an initial crack. The energy concentration along this direction is higher, leading to greater crack propagation in that direction. Conversely, less energy is distributed in the non-shaped charge direction, resulting in a significant advantage for crack propagation along the shaped charge direction. Under the influence of shaped charge direction blasting, the overall effect creates a crushing area, a crack area, and a vibration zrea.

The distribution of blast crack obtained from comparative test result shows that the numerical simulation result fully reproduce the fragmentation zone near the PMMA blast hole, as well as the initiation and propagation patterns of crack in both the shaped charge and non-shaped chage directions under the shaped charge blasting conditions. The propagation morphology of blast crack is consistent with the result of PMMA model test, confirming the accuracy of the established model and its numerical solutions. Further studies will continue to utilize this numerical model to investigate the evolution patterns of blast-induced crack under shaped charge blasting conditions.

## 4. Effect of borehole spacing in double-hole shaped charge blasting

### 4.1. Computational model

**Fig 3:**
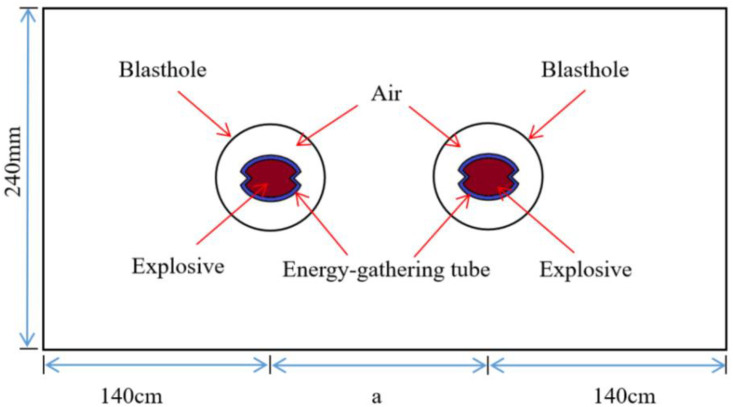
Calculation model of double-hole shaped charge blasting with different hole spacing.

To study the effect of borehole spacing on the propagation process of shaped charge blasting stress wave and the development of inter-hole crack, a double-hole shaped charge blasting calculation model with a borehole diameter of 85 mm was established based on practical field blasting experience. The distance a between the two boreholes was set to 80 cm, 90 cm, 100 cm, and 110 cm, respectively. The material parameters in the model were consistent with those in the previous section. The computational model was established as shown in Fig 3, employing a mapping mesh partitioning method. Non-reflective boundary conditions were added to the model periphery.

### 4.2. Stress wave propagation and crack propagation under different borehole spacing

**Fig 4:**
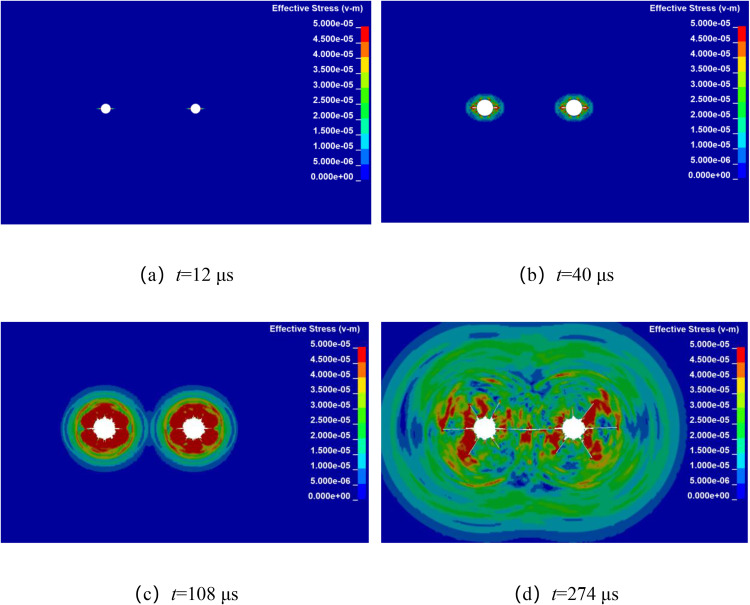
Crack propagation process with borehole spacing of 80 cm.

**Fig 5:**
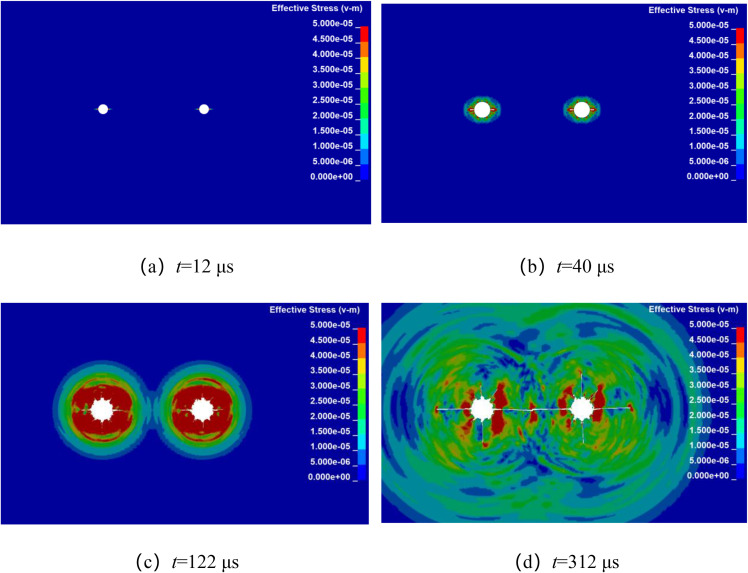
Crack propagation process with borehole spacing of 90 cm.

**Fig 6:**
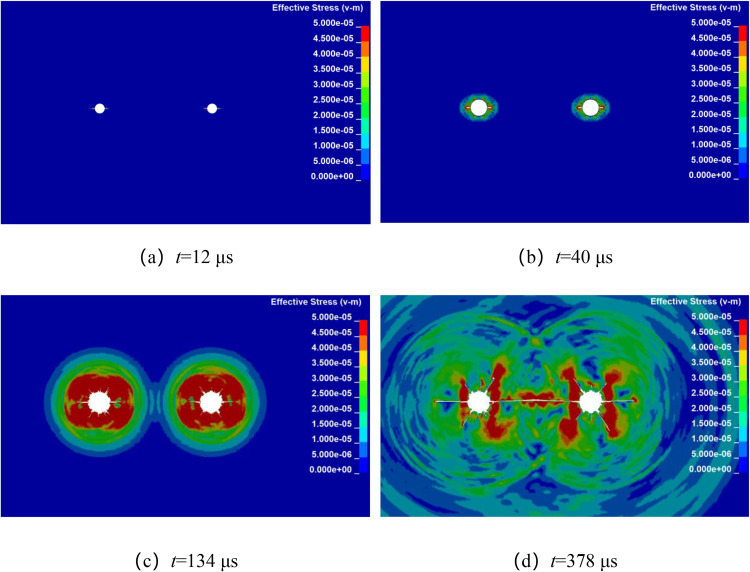
Crack propagation process with borehole spacing of 100 cm.

**Fig 7:**
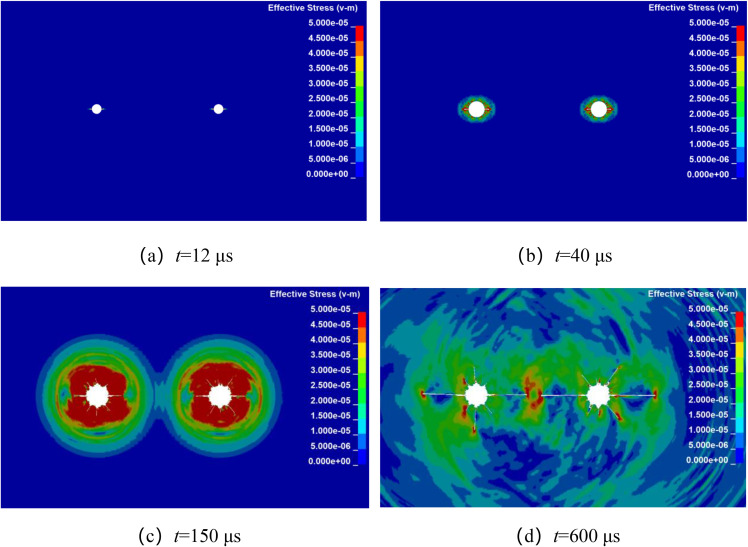
Crack propagation process with borehole spacing of 110 cm.

Numerical simulation was used to analyze the propagation of stress wave and the dynamic evolution of crack in double-hole shaped charge blasting with different hole spacing. By keeping all other factors constant, the explosives were detonated simultaneously at their centers, only the borehole spacing was altered. The stress wave propagation and crack dynamic evolution processes for double-hole shaped charge blasting with borehole spacing of 80 cm, 90 cm, 100 cm, and 110 cm are shown in Figs 4–7.

Fig 4 shows the propagation of stress wave and the dynamic evolution and propagation of crack during double-hole shaped charge blasting with a borehole spacing of 80 cm. It can be observed that at approximately 12 μs, the shaped charge jet penetrates the hole wall in the shaped charge direction, forming a guided initial crack. At approximately 40 μs, the stress wave propagates outward in an elliptical pattern, forming a certain range of fragmentation zones around the holes. In addition to the continuous expansion of crack in the shaped charge direction, crack also begin to form randomly in other directions. At approximately 108 μs, the stress wave from the two boreholes meet at their centers and begin to overlap, forming a stress concentration zone near the center of the line connecting the boreholes. The crack in both shaped charge and other directions continued to develop under the action of the explosive gas. At approximately 274 μs, the crack in the shaped charge direction formed a through crack along the line connecting the centers of the two boreholes.

Fig 5 shows the stress wave propagation and crack propagation process during double-hole shaped charge blasting with a borehole spacing of 90 cm. It can be seen that the stress wave propagation characteristics and crack propagation process during the blasting process are basically consistent with those of double-hole shaped charge blasting with a borehole spacing of 80 cm. At approximately 12 μs, the shaped charge jet penetrates the borehole wall in the shaped charge direction, forming a guided initial crack. At approximately 40 μs, the stress wave propagates outward in an elliptical pattern. As the borehole spacing increases, the time at which the stress wave from the two boreholes meet in the region along the line connecting the borehole centers is also delayed. At approximately 122 μs, the stress wave meet at the centers of the two boreholes, and at 312 μs, in the shaped charge direction formed a through crack along the line connecting the centers of the two boreholes.

Fig 6 shows the propagation of stress wave and the dynamic evolution and propagation of crack during double-hole shaped charge blasting with a borehole spacing of 100 cm. As can be seen from the figure, at approximately 12 μs, the shaped charge jet penetrates the borehole wall in the shaped charge direction, forming an initial crack. At 40 μs, the stress wave propagates outward in an elliptical pattern. At approximately 134 μs, the stress wave meet at the center of the two boreholes. At 378 μs, formed a through crack along the line connecting the centers of the two boreholes.

Fig 7 shows the propagation of stress wave and the dynamic evolution and propagation of crack during double-hole shaped charge blasting with a borehole spacing of 110 cm. As can be seen from the figure, at approximately 12 μs, the shaped charge jet penetrated the borehole wall in the shaped charge direction, forming an initial crack. At 40 μs, the stress wave propagated outward in an elliptical pattern. At 150 μs, the stress wave front met at the center of the two boreholes. At 600 μs, the crack propagation ceased but did not form a through crack.

By establishing numerical calculation models for shaped charge blasting with different borehole spacing, the propagation of stress wave and the dynamic evolution of crack were analyzed. It was found that the crack propagation mechanism of shaped charge blasting is basically consistent under different borehole spacing. The crack primarily propagate along the shaped charge direction, with fewer crack distributed in the non-shaped charge direction. The shaped charge blasting alters energy distribution, suppressing crack propagation in the non-shaped charge direction. As borehole spacing increases, the time at which stress wave meet between boreholes gradually delays. Comparing shaped charge blasting schemes under different borehole spacing, when the blast hole spacing is 80 ~ 100 cm, through crack formed in the shaped charge direction. However, when the borehole spacing is 110 cm, the crack does not form a through-crack in the shaped charge direction. This was primarily due to the combined effect of stress wave attenuation and insufficient driving capacity of the blast gas.

### 4.3. Effective stress of unit at different borehole spacing

**Fig 8:**
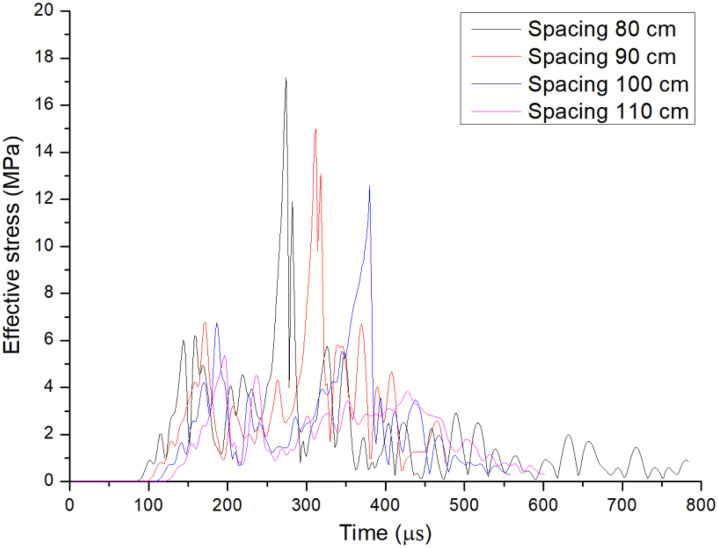
Time history curve of effective stress of shaped charge blasting with different hole spacing.

Based on the numerical model results of double-hole shaped charge blasting with different borehole spacing, the effective stress changes of the unit along the centerline of the boreholes were analyzed. The effective stress time history curves of the measurement point for double-hole shaped charge blasting with different borehole spacing are shown in Fig 8.

From the changes in effective stress at the center measurement point of the shaped charge blasting with borehole spacing of 80 cm, 90 cm, 100 cm, and 110 cm, it can be observed that the trend of effective stress changes under different borehole spacing follows a pattern of first increasing, then decreasing, followed by another increase before decreasing again. The first peak is primarily caused by the blast stress wave, while the velocity of the blast gas is slower than that of the stress wave. In the later stages, the damage primarily occurs under the quasi-static effect of the blast gas. As the spacing between boreholes increases, the peak effective stress at the measurement point also decreases. At a borehole spacing of 80 cm, the peak effective stress at the central measurement point is 17.2 MPa, at a borehole spacing of 90 cm, the peak effective stress at the central measurement point is 15.0 MPa; at a borehole spacing of 100 cm, the peak effective stress at the central measurement point is 12.6 MPa; and at a borehole spacing of 110 cm, the peak effective stress at the central measurement point later decreases to 3.8 MPa. An ideal blast borehole spacing should allow the main crack to form a through-crack without causing excessive damage to the inter-hole regions. When the borehole spacing is 110 cm, the main crack do not form a through-crack, indicating that the borehole spacing is too large. Conversely, too small a borehole spacing can lead to an increase in circumferential crack in the center regions of the boreholes, thereby affecting the blasting effectiveness.

## 5. Effect of detonation time difference in double-hole shaped charge blasting

### 5.1. Computational model

**Fig 9:**
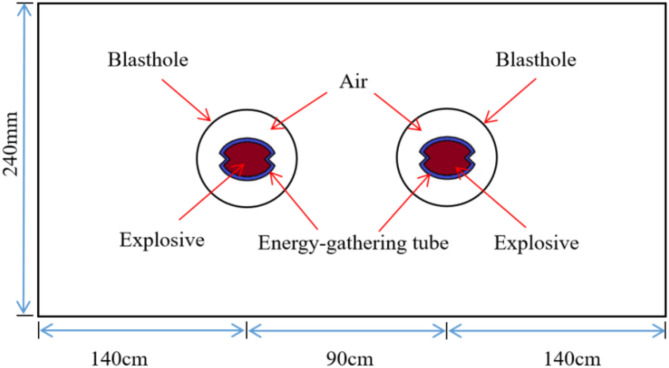
Numerical model of shaped charge millisecond blasting.

In practical engineering, it is difficult to ensure that all boreholes detonate simultaneously, even when using detonators of the same grade. During detonation, there is typically a certain time delay between boreholes. To investigate the effects of detonation time difference on the propagation of shaped charge blasting stress wave and the dynamic propagation of inter-borehole crack, a numerical analysis model for a double-borehole shaped charge blasting was established with a borehole diameter of 85 mm, a borehole spacing of 90 cm, with delay times of 20 μs, 50 μs, 80 μs, and 110 μs, respectively. The material parameters in the model were consistent with those mentioned earlier. Numerical calculation model as shown in Fig 9 was established, with non-reflective boundary conditions added around the model in the software.

### 5.2. Stress wave propagation and crack propagation in double-hole shaped charge blasting with differential detonation time difference

**Fig 10:**
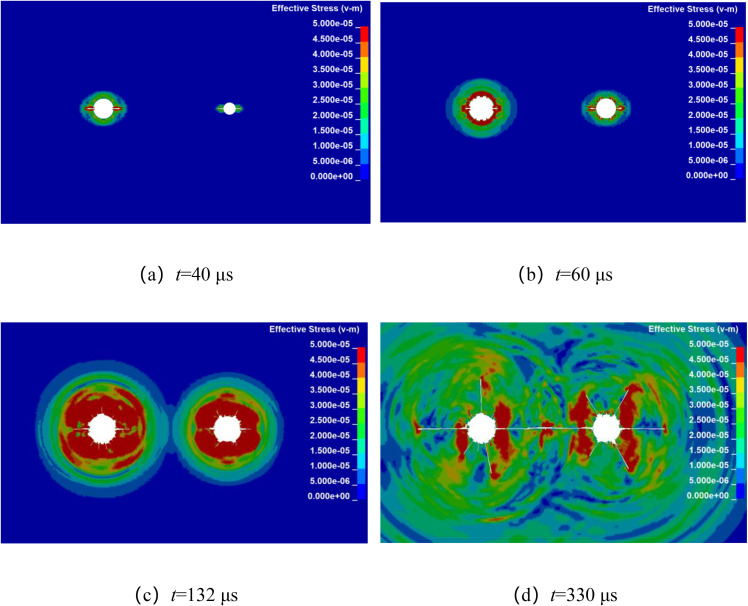
The millisecond time is 20 μs blasting crack propagation.

**Fig 11:**
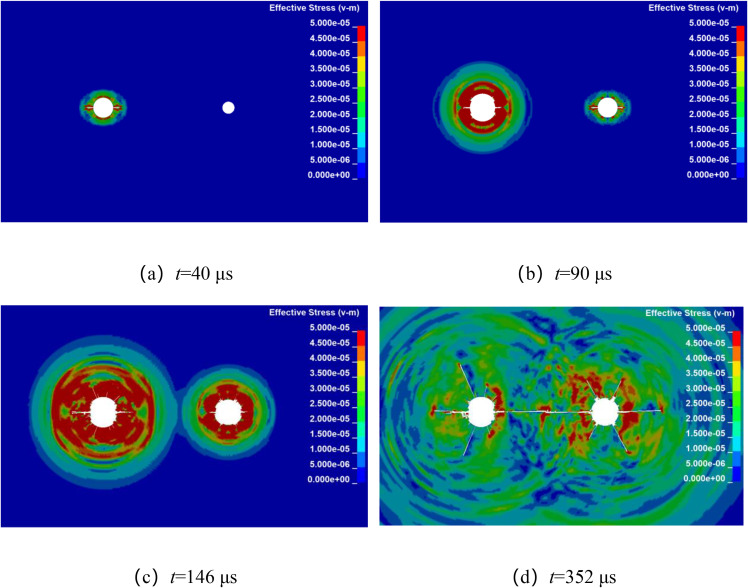
The millisecond time is 50 μs blasting crack propagation.

**Fig 12:**
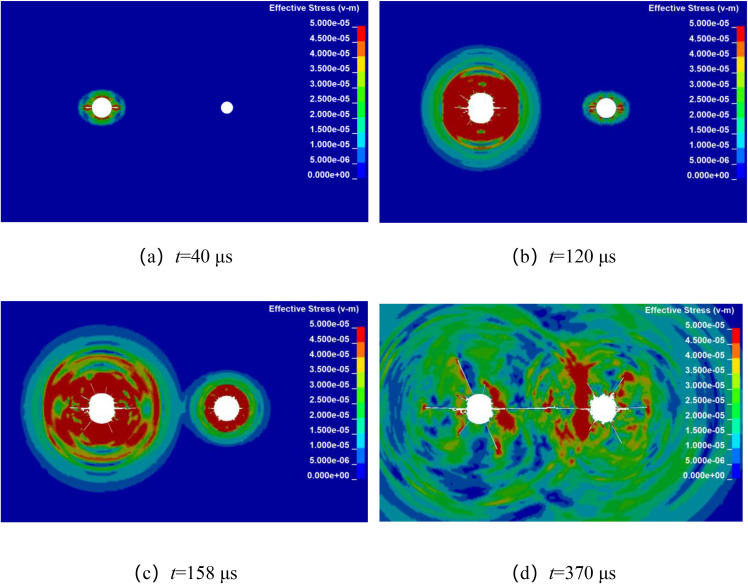
The millisecond time is 80 μs blasting crack propagation.

**Fig 13:**
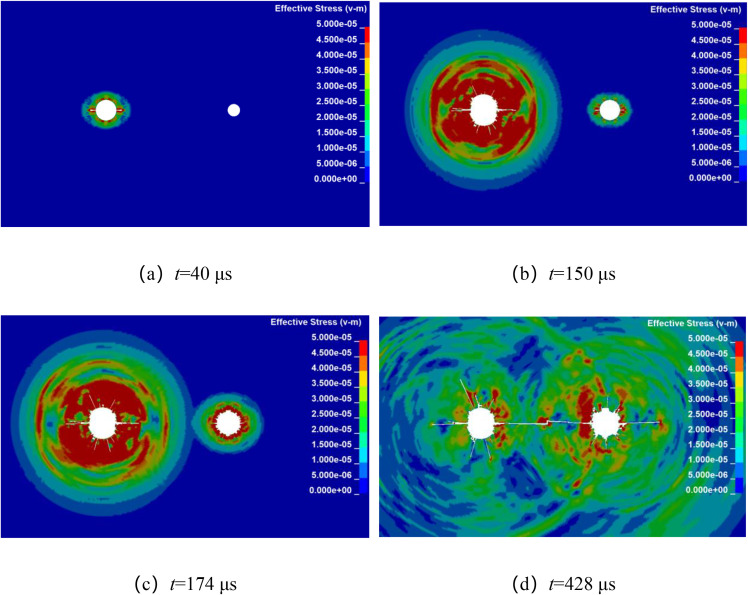
The millisecond time is 110 μs blasting crack propagation.

Numerical simulation was used to analyze the propagation of stress waves and the dynamic evolution of crack in double-hole shaped charge blasting with detonation time difference. By keeping other factors constant, with the left-side blast hole detonating first, while the detonation time of the right-side borehole is delayed by 20 μs, 50 μs, 80 μs, and 110 μs, respectively. The stress wave propagation and crack dynamic evolution processes for different micro-delay time in double-hole shaped charge blasting are shown in Figs 10–13. From the final crack propagation result in the figures, it can be seen that although through crack were formed in all cases, the propagation patterns of stress wave between boreholes and the crack propagation processes under micro-delay blasting exhibit certain differences.

As shown in Fig 10, during the stress wave propagation and crack propagation process with a microtime difference of 20 μs, it can be observed that during the initial stage of detonation at the primary detonation hole, the shaped charge jet penetrates the primary detonation hole to form a guide crack. Subsequently, the stress wave propagates outward in an elliptical pattern. The guide crack continues to expand under the combined action of the stress wave and explosive gas, similar to single-hole detonation. At 40 μs, the stress wave from the left-side primary blast hole is propagating outward in an elliptical pattern, while the right-side secondary blast hole has just penetrated to form the initial crack. At 60 μs, the stress wave from the right-side blast hole is propagating outward in an elliptical pattern. The early stages of both boreholes are similar to single-hole detonation. At 132 μs, the stress wave from both detonation boreholes meet and overlap. At 330 μs, formed a through crack along the line connecting the centers of the two boreholes.

As shown in Fig 11, during the stress wave propagation and crack propagation process with a microtime difference of 50 μs, at 40 μs, the stress wave from the left-side primary borehole is propagating outward in an elliptical pattern, while the stress wave from the right-side secondary borehole has not yet begun to propagate. At 90 μs, the stress wave from the right-side borehole is propagating outward in an elliptical pattern. At 146 μs, the stress wave begin to overlap, and at 352 μs, the crack between the boreholes becomes fully developed, formed a through crack along the line connecting the centers of the two boreholes.

As shown in Fig 12, during the stress wave propagation and crack propagation process with a microtime difference of 80 μs, at 40 μs, the stress wave from the left-side blast borehole is propagating outward in an elliptical pattern, while the right-side borehole has not yet initiated detonation. At 120 μs, the stress wave from the right-side borehole is propagating outward in an elliptical pattern. At 158 μs, the stress waves begin to overlap, and at 370 μs, the crack between the boreholes connect, formed a through crack along the line connecting the centers of the two boreholes.

From Fig 13, which shows the stress wave propagation and crack propagation process at a microtime difference of 110 μs, it can be seen that at 40 μs, the stress wave from the left borehole is propagating outward in an elliptical pattern. while the right-side borehole has not yet initiated detonation. At 150 μs, the stress wave from the right-side borehole is propagating outward in an elliptical pattern. At 174 μs, the stress waves begin to overlap, and at 428 μs, formed a through crack along the line connecting the centers of the two boreholes.

## 6. Conclusion

This paper establishes a numerical model for double-hole shaped charge blasting to analyze the mechanism of through-crack formation. It investigates the propagation process of shaped charge stress wave and crack propagation pattern under different borehole spacing and detonation time difference conditions. The following conclusions are drawn:

(1) Borehole spacing is a key factor in determining whether through-crack form. As the spacing between boreholes increases, the peak effective stress at the center of the connecting line between boreholes gradually decreases. Directional through-crack can form within a borehole spacing of 100 cm. However, when the spacing increases to 110 cm, the stress wave energy and explosive gas driving force become insufficient to form through-crack between the two boreholes.
(2) Detonation time difference influences the initiation and propagation sequence of crack between boreholes in shaped charge blasting by regulating the spatiotemporal distribution of the blast stress field. Under micro-delayed shaped charge blasting, the first-detonated hole creates a pre-stressed path for crack propagation. As the initiation time difference increases, the point of stress wave superposition between the two blast holes gradually shifts toward the later-detonated hole, and the crack connection point moves closer to it.
(3) Borehole spacing and detonation time difference distinctly affect crack propagation in shaped charge blasting. Excessively small spacing causes over-fragmentation, while overly large spacing prevents through-crack formation. An optimal micro-delay maximizes favorable conditions for crack propagation. Based on comprehensive simulation results, within the parameters defined in this study, a borehole spacing of 80–100 cm and a detonation time difference of 50–80 μs are recommended for optimal blasting design.
